# Fatigue Behavior of Cord-Rubber Composite Materials under Different Loading Conditions

**DOI:** 10.3390/ma17194771

**Published:** 2024-09-28

**Authors:** Julian Torggler, Martin Leitner, Christian Buzzi, Tobias Faethe, Heiko Müller, Eduardo Machado Charry

**Affiliations:** 1 Institute of Structural Durability and Railway Technology, Graz University of Technology, Inffeldgasse 25/D, 8010 Graz, Austria; 2 Siemens Mobility Austria GmbH, Eggenberger Straße 31, 8020 Graz, Austria; 3 GMT Gummi-Metall-Technik GmbH, Liechtersmatten 5, 77815 Bühl, Germany; 4 Institute of Solid State Physics, Graz University of Technology, Petersgasse 16, 8010 Graz, Austria

**Keywords:** cord-rubber composites, tomography, mechanical testing, fatigue design, numerical analysis

## Abstract

Cord-rubber composites are subjected to a wide range of loads in various applications. However, their fatigue behavior remains relatively under-researched. To address this gap, a set of representative specimens was developed, and a validated numerical model was employed to assess fatigue-relevant parameters. In this study, we present the results from two series of tests with different strain ratios (*R* values). One series was subjected to a pure pulsating tensile strain (*R* ~0), while the second series experienced an increased mean strain with an *R* ratio between 0.2 and 0.3. A direct comparison of the two series demonstrated that a higher strain ratio results in a longer service life. This is reflected in an increase in the slope (*k*) from 13 to 23, as well as an increase in the ultimate fiber strain from 8% to 11% at *N_d_* = 50,000 load cycles for a survival probability of 50%. Both series indicate a comparable scatter in the test results. This comparative analysis shows that the strain ratio significantly impacts the fatigue behavior of cord-rubber composite materials based on cyclic tests under different loading conditions. The findings of this study demonstrate the necessity of considering different load situations when evaluating or designing components.

## 1. Introduction

The assessment of fatigue in cord-rubber composites constitutes an intriguing field of research. Researchers have investigated the material properties, adhesion characteristics, and failure mechanisms of these composites, with the aim of improving the durability and performance of rubber products in various applications [1]. Due to the complexity of developing an air spring bellow for railway applications, the development process needs to be enhanced by investigating the material used. Based on component test results and investigations, a representative specimen geometry was developed, along with a testing methodology, to study the fatigue strength of different layups and new materials for new components at an early stage of development [2,3]. This approach allows for estimating the service life at critical states under biaxial loading with varying conditions.

Mars and Fatemi [4] conducted a comprehensive literature survey on factors influencing the fatigue life of rubber, categorizing them based on their mechanical loading history, environmental conditions, rubber compound formulation, and stress–strain constitutive behavior. This review provides a detailed overview of the various elements that impact the fatigue performance of rubber materials. Recently, these authors reviewed analytical approaches for predicting the fatigue life of rubber [5], with a particular focus on fatigue crack nucleation and growth in filled natural rubber. Moreover, Liu et al. [6] developed a probabilistic fatigue life prediction model for natural rubber components, taking into account the strain ratio effect, to simulate the relationship between mechanical properties and tensile fatigue life. Their analysis showed that the fatigue life of natural rubber follows a lognormal distribution. Luo [7] examined the fatigue prediction of filled natural rubber under both positive and negative R ratios, contributing to our understanding of how different stress ratios affect the fatigue behavior of filled natural rubber and providing essential data for predicting its durability and lifespan under varying loading conditions. A unified fatigue life prediction model for filled natural rubbers under uniaxial loads, proposed by Wang et al. [8], was applied. The effective tensile strain criterion can be applied for both positive and negative R ratios without additional fitting variables. A similar methodology was presented for the fatigue analysis of filled natural rubbers under positive and negative *R* ratios through a numerical investigation by Belkhiria et al. [9].

Assessing the fatigue in cord-rubber composites is crucial for the durability and reliability of rubber products. Although Fedorko et al. [10] focused on conveyor belts, their work highlights the importance of analyzing the structural integrity and failure modes of rubber-based products. By studying the failure mechanisms in cord-rubber conveyor belts, valuable knowledge can be extrapolated to improve the design and durability of rubber composites in various applications. The fatigue resistance of these composites is influenced by various factors, such as the type of material of the cord, its adhesion to rubber, and cyclic loading conditions. For instance, Tian et al. [11] pointed out the poor fatigue resistance and weak adhesion of a polyethylene terephthalate (PET) cord to rubber, underscoring the importance of cord-rubber adhesion in fatigue performance. Cutolo et al. [12] noted that elastomeric composites under shear loading can be affected by fatigue and delamination at the rubber–cord interface, characterized by adhesion, which compromises mechanical integrity. Liu and co-workers [13] focused on the stress and strain fatigue of unidirectional cord-rubber composites under pure tension loading, demonstrating the importance of understanding fatigue properties under different loading conditions. Tian et al. [14] investigated the fatigue properties of steel cord-rubber laminates under pure tension loading, shedding light on material behavior under cyclic loading. They also explored the dynamic mechanical properties of a nylon-like polyester tire cord through a dynamic mechanical analysis and fatigue testing [11].

The stress ratio, which is the minimum stress divided by the maximum stress, is a key factor in how composites behave under fatigue loading [15,16,17,18]. Research shows that the stress ratio significantly affects the fatigue life of composites. Different stress ratios can result in different fatigue behaviors [16,17]. For example, increasing the stress ratio from 0.1 to 0.3 improves the fatigue life of glass fiber-reinforced plastic composites at all load levels [17]. The effects of the stress ratio on the fatigue behavior of composites have been studied in various situations, including studies investigating pure tension fatigue behaviors [16] and mode I fatigue delamination growths [15]. These studies have shown that the stress ratio has a major effect on the fatigue behavior of composite materials.

Many damage parameters have been used to predict the fatigue life of rubber, with strain measurements often serving as key damage indicators in rubber fatigue studies [4,19,20]. Consequently, a strain-based approach is adopted in this study, as its importance to damage prediction has been recognized for cord-rubber composites: the object of inquiry in the present study. In particular, the influence of the fiber strain ratio (minimum to maximum fiber strain) on fatigue strength is examined. The maximum fiber strain is used as a damage indicator, because it has the best correlation with damage. Understanding the effects of the strain ratio is critical to predicting fatigue life, damage progression, crack initiation, and crack growth in composites and will help to develop more accurate fatigue models and design guidelines.

This paper presents two series of specimen tests with different loading conditions which demonstrate the influence of the strain ratio on the composite material. A specially developed representative specimen and a test methodology derived from railway air spring applications are used for this purpose. This approach facilitates the study of the fatigue behavior of cord-rubber composites using specimen tests, thus avoiding the need for time-consuming and expensive component testing.

This paper introduces a novel approach by comparing specimen tests under different loading ratios, which represent typical loading scenarios for railway air spring bellows. The fatigue life evaluation method offers an efficient way to estimate the service life, using the maximum fiber strain as a key predictor. A comparison of S/N curves for pure pulsating tensile and pulsating tensile specimens clearly demonstrates a significant impact of the strain ratio on fatigue life.

## 2. Materials and Methods

**Figure 1:**
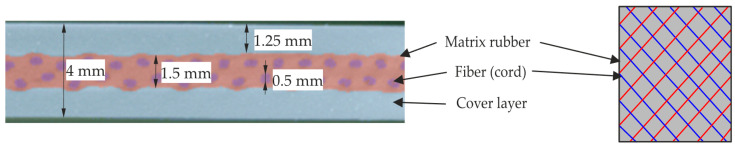
Cord-rubber composite structure: micro-computed tomography section (**left**) and sketch of front view of fiber arrangement (**right**).

**Figure 2:**
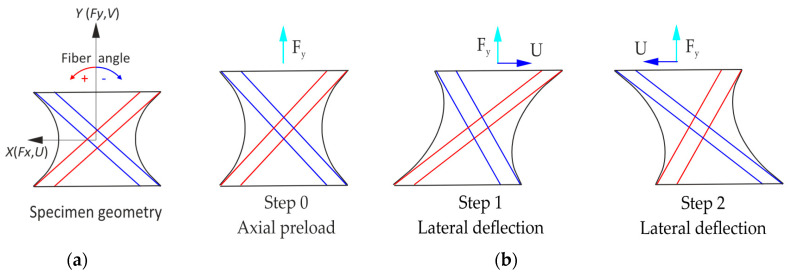
(**a**) Representative specimen geometry and coordinate system and (**b**) test procedure on the flat specimen.

In general, a fiber composite consists of two main components, the reinforcing fibers and the embedding matrix. These components have different mechanical properties, which are combined in a composite material to create new properties. In this study, we examine a composite made of cord fibers (polyamide) and rubber (a natural rubber compound with a hardness of 58 Shore A). Figure 1 illustrates the structural composition of the composite material under investigation, showing the matrix, fiber, and cover layers in sections as well as the fiber arrangement in a front view. As marked in Figure 1, the thread in question had a diameter of around 0.5 mm and was composed of two twisted polyamide (PA) strands with a tensile strength of approximately 950 MPa and a modulus of elasticity of approximately 6 GPa [2]. The developed material specimen featured a test area of approximately 60 × 78 mm, as illustrated in Figure 2a.

The following subsection briefly summarizes the key findings from previous work, particularly from the sources [2,21,22]. It introduces the specially developed representative specimen and the test methodology derived from the application of railway air springs. Next, the methodology for the damage analysis in the tests is presented through a representative example. Finally, the numerical model used for a fatigue assessment of the specimen is described and validated using measured data.

### 2.1. Representative Specimen Design and Testing Methodology

A material specimen was developed to investigate its fatigue strength and to assess damage behaviors [1,23]. The specimen’s geometry, derived from the air spring bellow in railway applications, was designed to study the predominant damage mechanism, delamination [2,24]. The stresses in the specimen were based on typical loading situations experienced by the component. Several specimen concepts were compared, with the flat specimen meeting the requirements most effectively and, therefore, being implemented. The Arcan-like specimen was developed according to specific criteria, focusing on clamping, manufacturing efforts, and the mapping of relevant damage mechanisms. These considerations led to the implementation of the flat specimen. The mechanical properties of the material were investigated using the testing standards developed for the fiber composite [2].

Rubberized cord layers are available in mats containing fibers embedded in natural rubber at a defined distance of 0.95 mm. The rubber-cover layers and the cord layers were cut to size and were placed together with clamping plates in a negative mold. The specimen consisted of at least two layers of rubberized cord mat and rubber-cover layers, which were vulcanized together at 160 °C for 20–30 min. The process parameters for specimen manufacturing were selected to achieve a state of vulcanization as similar to that of the air spring bellow as possible.

Failures detected during bellow testing coincided with the stress hotspot evaluation, identifying delamination as the dominant damage mechanism [24]. A numerical model of the bellows was used to evaluate the stress hotspot within the composite material. The specimen geometry was designed to represent this area, mapping the relevant damage mechanisms observed during service life testing. Figure 2a shows the representative specimen geometry and defined coordinate system. More detailed information can be found in [2].

The fiber angle ranged from 0 to 35 degrees with respect to the *Y* axis, accommodating up to eight fiber layers. The cord layers served as the strength members, encased in the elastomer matrix, which stabilized the geometry and ensured correct fiber positioning. Vulcanized steel plates were used at the connecting points for clamping and to guarantee accurate geometries and positions.

The primary loads acting on the air spring bellows were internal pressure and lateral displacement. Based on these loading conditions, the flat specimen was loaded in a comparable manner. Figure 2b illustrates the test procedure that the flat specimen was subjected to. Initially, an axial preload was applied and maintained at a constant level throughout the test (with a deviation of less than 2%). Subsequently, a sinusoidal lateral deflection was applied with a constant amplitude.

The failure criterion was defined as a 20% increase in mean axial displacement, based on a comparable criterion in the component test. The test frequencies ranged from 1 to 3 Hz, limited by a maximum surface temperature of 50 °C, to avoid a significant change in the material properties. A global and a local approach to evaluating fatigue testing are presented in [21]. With the help of a simplified analytical approach, a first master S/N curve for the evaluated tests was developed. In this paper, a computational method is presented, and a numerical model of the specimen is created and evaluated.

The damage mechanisms were investigated in detail at the macro level using a layup equivalent to that of the air spring bellows. The specimen exhibited the same types of damage observed in the air spring bellows. The methodology for damage analysis is discussed in the following section.

### 2.2. Fracture Pattern Investigation

The manner in which a material responds to stress can vary depending on specific circumstances. The initial step in damage analysis is visual inspection. For a more comprehensive examination, micro-computed tomography (µCT) scans are employed on selected specimens to identify damage in composite materials without causing any damage to the specimens. The primary challenge in this process is the small density difference between rubber and polyamide fibers. Due to the trapped air in the investigated cord’s thread, this difference was recognizable, bearing almost the same material density as the matrix; the manufacturer specifies 1.13–1.16 g/cm^3^ for the elastomer matrix and 1.14 g/cm^3^ for that of the polyamide.

A laboratory-based µCT device (UniTOM XL, Tescan Orsay Holding, Brno, Czech Republic) was utilized for the 3D structural characterization of the cord-rubber composite. This device was equipped with an amorphous silicon flat panel detector from Varex. The X-ray acceleration voltage was set to 92 kV, and the target current was 370 μA. To harden the beam, a 1.5 mm-thick aluminum filter was used. Each scan acquired approximately 4283 projections over a 360-degree angular range. Each projection had an exposure time of 0.450 s, with two averages and no binning. The camera’s pixel matrix and a geometrical magnification of approximately 4.4 yielded an isotropic voxel size of 34 μm. The field of view for each specimen was approximately 97 mm × 67 mm. After a scan reconstruction using the Panthera 1.4.4 software (TESCAN XRE, Brno, Czech Republic), a data analysis was performed with the Dragonfly software, Version 2024.1, for Windows.

A generic pre-trained segmentation model, specifically, U-Net with a depth level of 5 and an initial filter count of 64, was employed using the Segmentation Wizard in Dragonfly. Based on the user’s input, the specimen is divided into its components, i.e., fibers, matrix, protective rubber, and air. Dragonfly’s Segmentation Wizard then automatically assigns a material to each part of the specimen based on a few examples provided by the user. By adding additional training data to problematic regions, models can be fine-tuned. Segmentation enables the identification of cracks and fractures, a volume analysis of defects, and an analysis of material homogeneity. Advantages of the AI model include creating a single model for different components, reducing misclassifications, and resolving edges with greater precision.

**Figure 3:**
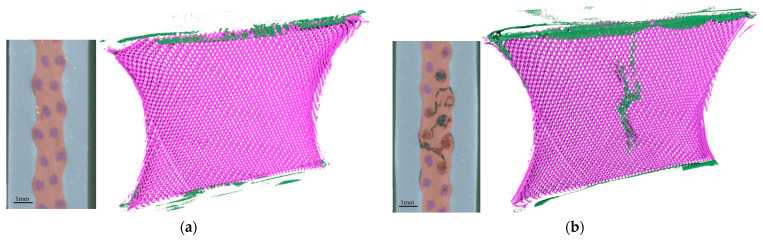
µCT evaluation showing section and segmentation results: fiber (purple), matrix (pink), surface layer (gray), and air (green). (**a**) Specimen with minor cracks and lightly damaged filaments and (**b**) specimen with significant damage and broken filaments.

As in the initial optical analysis, two principal types of damage can be identified. Figure 3 presents a comparison of two damage patterns immediately following the attainment of the defined damage criteria. This illustration shows a section and the fiber arrangement in the specimen with air inclusions. Specimen (a) exhibits no externally visible damage. However, delamination between the matrix and the rubber-cover layer is discernible through segmentation, along with numerous smaller defects within the specimen. Delamination typically represents the most significant damage in terms of volume, while fiber damage on the interior and separate inclusions usually results in smaller partial volumes. Specimen (b) is a severely damaged component with externally visible damage. Torn fibers are clearly visible within the specimen, causing the irregularities on the surface. Air inclusions and partial layer separation (delamination) can also be clearly identified using segmentation.

The different failure behaviors can also be observed during test runs. In case (a), where no damage is visible on the specimen surface, a test can be continued once the defined criterion has been reached, showing a continuous decline in the shear force until the failure criterion is reached. The second type of damage (b) is visible on the specimen surface and results in an abrupt failure, making it difficult to continue a test. This abrupt failure is evidenced by a sharp decline in shear force. The data curves indicate either a lateral drift or a constant lateral load center position, in accordance with the requirement of force symmetry. A Weibull analysis of tests with identical parameters indicates that the two discernible failure modes can be considered collectively [22].

Specimens with defects resulting from production issues, such as incorrectly inserted plies, significant asymmetries, and poor vulcanization, were excluded from this analysis. Additionally, the test bench and controller errors, primarily due to operational issues, were not included. Furthermore, tests that did not reach the failure criterion after a defined number of cycles (2 × 10^6^) were not considered either. Tests below 1000 cycles were excluded from further evaluation due to a lack of practical relevance, with 5000 to 10,000 cycles typically being the lower limit [21].

To ensure the transferability of the results to the component level, a numerical model of the specimen was used to evaluate damage-relevant parameters. A suitable numerical modeling technique is presented in the following subsection. Based on a model for air spring bellows [25], the numerical modeling technique was applied to the specimen.

### 2.3. Calculation Procedure: Numerical Modelling of Specimen

The numerical investigation of the specimen properties was conducted using the MSC Marc Mentat 2022 Feature Pack 1 software, which is designed for non-linear application calculations employing the finite element (FE) method.

For the air spring bellow, a three-dimensional simulation was utilized [22]. The cord layers were simplified using so-called rebar (solid) elements. Hex-8 solid elements were also used to model the specimen. Element type 146 for superimposed rebar elements represents an isoparametric, three-dimensional, eight-node empty brick with directional reinforcement elements inserted. These layers represent the cord layer properties at the nodes, with the nodes of the superimposed rebar element being used in conjunction with the underlying elastomer element. The relative position of each layer in the thickness direction of the rebar element can be specified, with the layers evenly distributed across the thickness in the model created. Rebar elements are unidirectional, and the properties of the cord layer were determined by the fiber cross-sectional area (0.25 mm^2^), fiber spacing (0.95 fibers/mm), angle (depending on confection), and material properties [22].

The material properties of the cord and the elastomer matrix in the FE model are defined differently. A stress–strain relationship for the cord was developed, as described by two stiffness ranges based on [26]. In these areas, the stress–strain relationship is linear [22]. In the first range, from 0 to 2.4% strain, an elastic modulus of 800 MPa was assumed and above that, an elastic modulus of 1750 MPa.

For the elastomer stress–strain behavior, hyperelastic material models are commonly used [26]. However, dissipative effects were not considered, leading to coinciding loading and unloading paths. The “Yeoh model” [27] was employed for the finite element analysis. The elastic potential (*W*) is described by the following formula:

W = C_10_ (I_1_ − 3) + C_20_ (I_1_ − 3)^2^ + C_30_ (I_1_ − 3)^3^, (1)

where the constants C_10_ = 0.4736 MPa, C_20_ = 0.0196 MPa, and C_30_ = 0.00056 MPa characterize material parameters. I_1_ represents the first-order deformation invariant. Element type 84 for elastomers is an eight-node isoparametric element with an additional ninth node for pressure, allowing for material incompressibility.

The clamping plates were modeled as rigid bodies, with a surface added and “bonded” to the composite material. The load was applied via the control nodes of the geometric bodies, with one side being fixed and the other subjected to a load. Initially, an axial preload was applied, followed by a lateral deflection under a constant axial preload, in accordance with the test procedure on the flat specimen, as illustrated in Figure 2.

**Figure 4:**
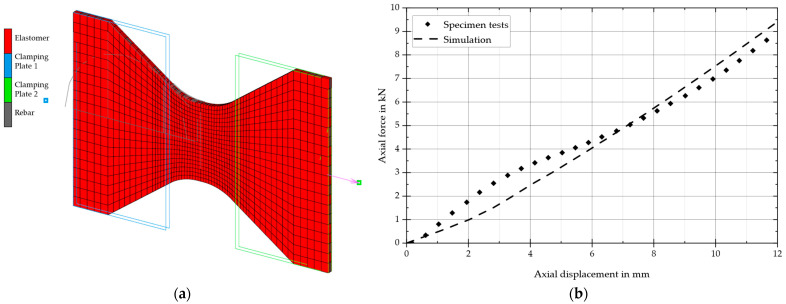
(**a**) FE model of the specimen and (**b**) evaluation of one load cycle.

The specimen was modeled in the FE software, as illustrated in Figure 4a. The initial step involved comparing the measured axial force under a purely uniaxial load with the simulated force, as illustrated in Figure 4b for a specimen with a 15-degree fiber angle and four plies.

The air spring bellow model was used to define a range of load scenarios for the flat specimen. The fiber strain showed the best correlation with the analyzed specimen tests and was used as a damage evaluation indicator [22]. Fiber strain is evaluated at the nodes identified as hotspots, focusing on the central node in the inner layer. For each specimen test, a finite element model must be evaluated at the specified load level with the designated fiber angle.

**Figure 5:**
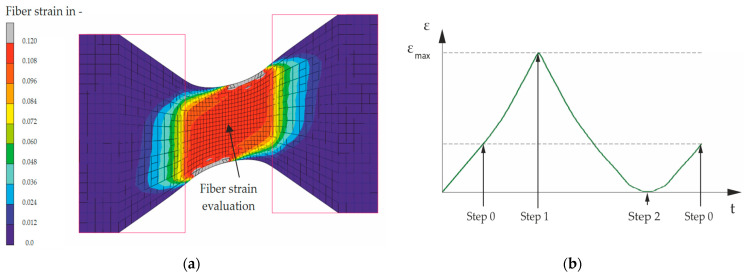
(**a**) Evaluation of fiber strain, location of evaluation, and (**b**) one cycle of fiber strain: step 0—axial preload, step 1—lateral deflection (loading), step 2—lateral deflection (unloading).

Figure 5a shows an example of a fiber strain in a deflected specimen subjected to an axial load (*F_y_*) of 4 kN and a lateral deflection amplitude of 15 mm. The specimen exhibited a uniform strain distribution on the fibers, with the grayed-out-edge areas indicating excessive stress due to the geometry and a relieved area on the opposite side. Similar to the air spring model, a single cycle was simulated. Figure 5b illustrates the evolution of the fiber strain in a representative load scenario, with the steps being marked according to the test procedure outlined for the flat specimen in Figure 2. The strain was localized and evaluated at the center of the same layer of the specimen for each load case.

The advantage of using superimposed rebar elements lies in their ability to facilitate straightforward alterations to the rebar layer distribution, ease of hotspot evaluation, and relatively short modeling times. Verification of the FE models was performed by comparing the force-over-distance behavior of the FE models with the test results as well as the surface strain measurements for material-model validation, as discussed in the next section.

### 2.4. Non-Contact Strain Measurements by Means of Image Correlations

When measuring the real specimen, the tensile force and displacement are recorded by default. These global measurements do not allow for direct insights into local effects. Therefore, an additional measurement method that can detect this effect is needed. One possible approach is to analyze the deformation image of the specimen. In the MSC Marc Mentat 2022 Feature Pack 1 simulation software, strain can be read out in the respective principal directions and as a maximum value, allowing for a comparison of the specimen’s surface deformation with that in the simulation.

An optical (camera-based) deformation measurement method is a gray value correlation (also known as an image correlation). During the deformation process, a camera records the stochastic pattern applied to the specimen surface. The displaced pattern in the recorded camera image is identified, and the displacements on the component surface are calculated from the measured pixel coordinates. These methods measure the true (logarithmic) strain on the surface by tracking the points of the surface pattern.

**Figure 6:**
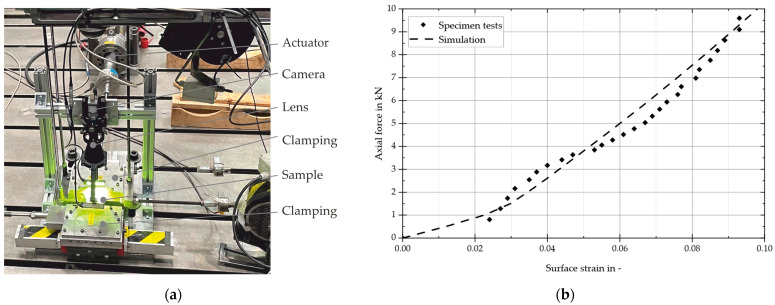
(**a**) Measurement setup for surface strain measurements and (**b**) axial force in relation to axial strain.

Figure 6a depicts the detailed setup of the camera and lens with LED illumination. The camera used was a Basler acA2000-50gm industrial camera, which can deliver 50 frames per second with a resolution of two megapixels. The TechSpec telecentric lens used is suitable for 1/2” and 2/3” sensors. These lenses are designed for test applications and feature a simplified, non-focusing mechanical construction and an adjustable aperture with a locking screw. This setup was calibrated using a glass calibration plate, and a pixel edge length of 35 μm was measured.

In the first step, the force and displacement signals of the tensile testing machine and the camera images were analyzed. These analyses enabled a comparison between the tensile test and the numerical model. Figure 6b illustrates the axial force in relation to the strain. A comparison between the simulation model and the measurement demonstrated a satisfactory level of agreement, particularly in the evaluation-relevant range with up to approximately 10% elongation.

## 3. Results

The results of the specimen’s fatigue tests are presented in this chapter. Two series of specimens were subjected to experiments, each with different axial preloads (*F_y_*) and lateral displacement amplitudes (*U*). The load situations were designed to mimic the conditions experienced by air spring components, resulting in mean strains of approximately 4–7% and strain amplitudes of approximately 3–5%.

The specimens of series 1 had fiber angles of ±5, ±15, ±25, and ±35 degrees and four layers. These specimens were tested under a pure pulsating tensile stress, with a strain ratio (*R*) of approximately 0. The specimens of series 2 had the same fiber angles of ±5, ±15, ±25, and ±35 degrees but with only two layers. These specimens were tested under load conditions resulting in a strain ratio (*R*) of approximately 0.2 to 0.3. The specific load conditions were selected deliberately, based on the understanding that the stress ratio is essential to the behavior of composites under fatigue loading [15,16,17,18]. This study uses a strain-based approach, because strain measurements often serve as a key damage indicator in rubber fatigue [4,19,20].

### 3.1. Series 1: Experimental Results

**Figure 7:**
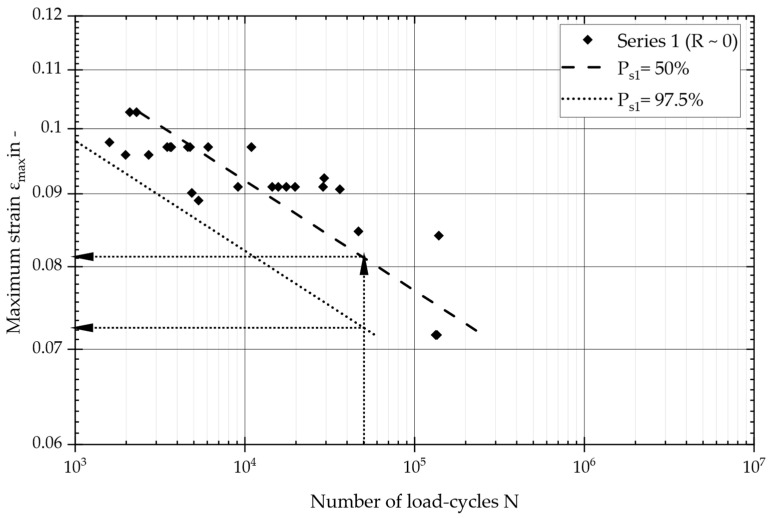
Master S–N curve and maximum fiber strain against number of load cycles: evaluation of the tests of series 1, covering tests with a pure pulsating tensile strain (R = 0).

As previously stated, the primary objective of this series of tests was to investigate the effects of pure pulsating tensile loading. Therefore, strain ratios of approximately 0 were deliberately selected within the range of pure pulsating tensile stresses. The endured load cycles ranged from 1 × 10^3^ and 1 × 10^5^. Using the presented numerical model, all these load cases were calculated with the specified fiber angles, and the local fiber elongation at the center of the specimen geometry was evaluated. The maximum fiber strain is plotted against the number of load cycles in Figure 7, which also includes the results of a statistical evaluation, in accordance with DIN 50100 [28]. As outlined in the research conducted by Liu et al. [6], the lognormal distribution was employed to describe the fatigue strength of the material.

The design curve was defined at *N_d_* = 50,000 load cycles (based on DIN EN 13597 [29]) and with a survival probability of *P_s_* = 97.5%. Figure 7 shows the statistically evaluated master S/N curve, covering all test results, utilizing the maximum strain (*ε_max_*). The S/N curve parameters resulted in a slope of *k* = 12.9 and an ultimate fiber strain of *ε_max_* = 8.1% at *N_d_* = 50,000 load cycles for a survival probability of 50%. The statistically evaluated scatter band was adequate, indicating a value of *T_s_* = 1:1.16. This design curve could be further used for a comparison with the second series of tests.

### 3.2. Series 2: Experimental Results

**Figure 8:**
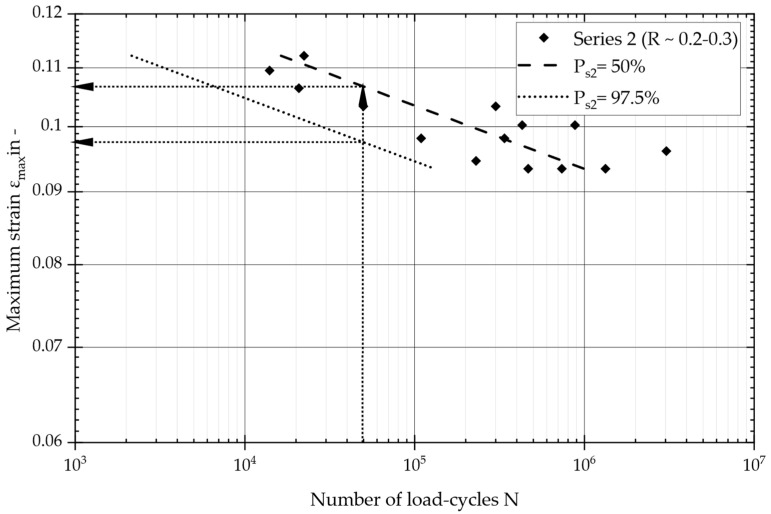
Master S–N curve and maximum fiber strain against number of load cycles: evaluation of the tests of series 2, covering tests with a pulsating tensile strain (R = 0.2–0.3).

In the second series of tests, the focus was on maintaining a certain tension in the fiber within the range of pulsating tensile strain (*R* ~0.2–0.3). A numerical model of the specimen was employed to evaluate the maximum fiber strain at the center of the specimen, which is plotted over load cycles in Figure 8. The test results are presented in terms of load cycles, with values ranging from 1 × 10^4^ to 1 × 10^7^, including the results of a statistical evaluation, in accordance with DIN 50100 [28].

As before, the design curve was defined at a load cycle threshold of *N_d_* = 50,000 (based on DIN EN 13597 [29]), with a survival probability of *P_s_* = 97.5%. Figure 8 shows the statistically evaluated master S/N curve, covering all test results, utilizing the maximum strain (*ε_max_*). The S/N curve parameters indicate a slope of *k* = 22.5 and an ultimate fiber strain of *ε_max_* = 10.7% at *N_d_* = 50,000 load cycles for a survival probability of 50%. The statistically evaluated scatter band was adequate, indicating a value of *T_s_* = 1:1.12. The subsequent section directly compares and discusses the two obtained S/N curves.

## 4. Discussion

**Figure 9:**
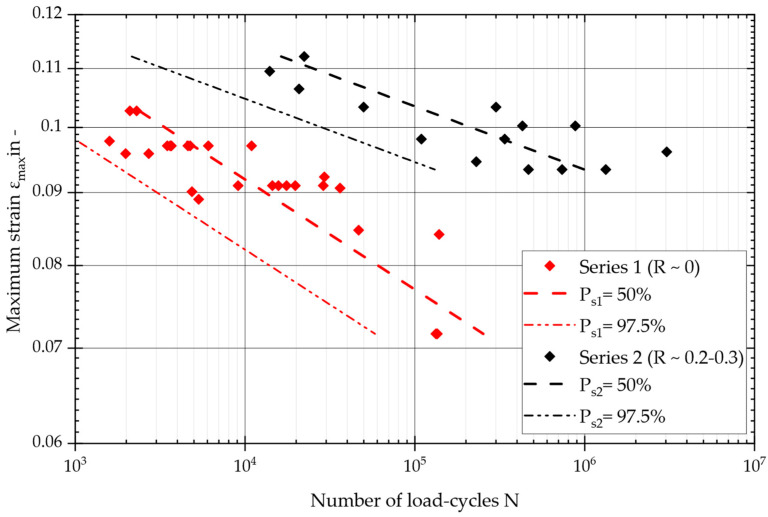
Master S–N curves and a comparison of specimens tested with a pure pulsating tensile strain (series 1) (R = 0) and a pulsating tensile strain (series 2) (R = 0.2–0.3).

The statistically evaluated design S/N curves from the specimen tests can now be consulted to compare and assess the influence of the strain ratio on the test results. Specimen tests on different confections (four and two layers and 5–35 degrees in fiber angle) show a clear strain ratio dependence. As has been demonstrated for other composites [16,17], this is also the case here for the material under investigation. Figure 9 displays the two sets of tests in one diagram, where the maximum fiber strain obtained from the FE simulation is plotted against the load cycles. At this stage, we can evaluate the expected life for different survival probabilities. This diagram shows the probability of survival (*P_s_*) at 50% and 97.5%. The tests of series 1, with a pure pulsating tensile strain (*R* ~0), showed significantly fewer load cycles than those of series 2, with a pulsating tensile strain (*R* ~0.2–0.3). It is not possible for fibers to absorb compressive loads, as they are unable to transmit compressive forces. Pure tensile swelling results in the fiber becoming tensionless, which is considered damaging to the interface between the fiber and the matrix and is likely to favor delamination [30,31,32].

The scatter in the two test series is within a similar range, with *T_s_* = 1:1.16 for series 1 and *T_s_* = 1:1.12 for series 2. Series 1 comprised 23 valid specimen tests, while series 2 included 14 valid tests. Given the small number of tests, any statistical analysis of these data should be treated with caution. The higher slope of the S/N curve and the higher maximum fiber strain at the design point indicate a significantly longer service life at *R* ~0.2–0.3. Series 1 achieved fewer than 1 million cycles at high deflections, while series 2 focused on higher cycles at lower deflections. These load situations, derived from the air spring component, are relevant to real operations. However, it should be noted that load collectives from operational measurements or multibody simulations were not considered. Both test series applied a block load of a constant amplitude, based on a symmetrical load, without considering aging (laboratory conditions) or stochastic loads. Depending on the requirements, the following limitations and general conditions of the presented methodology have to be considered.

Two series of tests, one with two layers and another with four layers, were compared under different strain ratios (R values). The impact of the number of layers on the selected specimens, using load cases from the other series, was found to be either negligible or so variable that it was lost in the scatter of the tests. Each series contained the same number of specimens for the individual angle assemblies. Series 1 (four layers) had more valid tests, with 23 tests being analyzed compared with the 14 tests of series 2. The relatively small number of tests should be considered when interpreting the results.

Various approaches can be used to numerically calculate the local variables of fiber composites [1,23]. The mesoscale model presented here was used to calculate the fiber strain at the center of the specimen. The fiber angle of the specimen was assumed to be specified in the model, understanding that it may deviate due to the manufacturing process. This deviation due to asymmetry is reflected in the scatter of the test data. Specimens with a force asymmetry greater than 10% at the geometric center of the specimen were not considered.

To compare the simulation with the test, a non-contact strain measurement method was employed by means of image correlations. The outcomes of the force and non-contact strain measurements were compared with the simulation results, showing good agreement in the area of interest. The numerical model was calibrated using the measurements taken of several specimens with different confections. The results presented in this study demonstrate the effectiveness of this procedure.

The limitations of the optical two-dimensional surface strain measurements are briefly outlined here. To eliminate lens distortion, a telecentric lens was selected and calibrated. Non-telecentric lenses can cause a barrel-shaped distortion or image offset. Changes in the distance between the camera and the specimen, triggered by camera movements or vibrations (e.g., caused by a passing person), can also introduce errors. A cosine error is caused by the tilting of the specimen, where it tilts around the center point but not at the edge. This effect causes a deviation of −0.06% with a two-degree tilt. However, the overall deviations for a measurement range of up to a 10% strain are considered negligible.

To assess the deviations in the symmetry of the specimens, it is possible to use the deviation from the geometric center in the steady-state position to quantify this effect. The asymmetry of the specimen can be evaluated by considering the geometric position of the center of vibration clearance in the steady-state position. However, no practical and straightforward method could be identified, and, therefore, this discrepancy was accepted. For simplicity and general applicability, the specified fiber angle was used in the numerical simulation.

As mentioned, both the representative specimen geometry and the biaxial testing method are novel in this field. While previous studies have primarily focused on the uniaxial fatigue behavior of similar materials [13,16,17], this study uses two independently controlled axes to adjust the loading ratio. By applying this approach to the actual bellow layup, rather than to individual layers or fibers [12,23,32], we can effectively investigate various layups and material combinations.

## 5. Conclusions

Based on existing component tests, a test methodology was applied to a specially developed, representative specimen. Various loading conditions were analyzed, and a failure criterion was defined as a 20% increase in the mean axial displacement. All specimens were visually inspected, and selected specimens underwent a µCT analysis to identify the type of damage inflicted. Two different main damage patterns were present, resulting in a comparable service life. A numerical model of the specimens was validated through measurements of the surface strain via 2D-image correlations.

A local evaluation of the fiber strain generated master S/N curves. An investigation of different load cases with an axial preload (*F_y_*) (resulting in a mean fiber strain of 4 to 7%) and lateral deflection (*U*) (causing fiber strain amplitudes of 3 to 5%) revealed a clear dependence of the service life on the strain ratio. This finding aligns with those of previous studies, confirming the detrimental effect of fiber becoming tensionless. It was found that pulsating tensile stresses with a strain ratio of *R* > 0.2 significantly increased the service life of the fiber composite material for the investigated load cases. Both the slope (*k*) (from 13 to 23) and the level of the maximum tolerable strain (from 8 to 11%) increase in the S/N diagram as the *R* ratio increases from *R* ~0 to *R* ~0.2–0.3.

Further research should explore the influence of variable load amplitudes (VALs) and possible sequence effects, as well as center load effects (asymmetric loads). This could lead to the derivation of damage-accumulation hypotheses and allowable damage sums. Also considering aging behaviors could provide a holistic fatigue-strength-prediction method, which would be useful for the detailed design of future components using the investigated composite material.

Understanding the fatigue behavior of cord-rubber composites has many practical benefits in terms of product reliability, safety, and performance. Economically, it leads to cost saving by reducing maintenance and replacement costs.

## Data Availability

The raw data supporting the conclusions of this article will be made available by the authors on request.
